# Son of a Lesser God: The Case of Cell Therapy for Refractory Angina

**DOI:** 10.3389/fcvm.2021.709795

**Published:** 2021-09-06

**Authors:** Beatrice Bassetti, Erica Rurali, Elisa Gambini, Giulio Pompilio

**Affiliations:** ^1^Unità di Biologia Vascolare e Medicina Rigenerativa, Centro Cardiologico Monzino-Istituto di Ricovero e Cura a Carattere Scientifico (IRCCS), Milan, Italy; ^2^Oloker Therapeutics S.r.l., Bari, Italy; ^3^Dipartimento di Scienze Biomediche, Chirurgiche e Odontoiatriche, Università degli Studi di Milano, Milan, Italy

**Keywords:** refractory angina, cell therapy, meta-analyses, recommendation, guidelines

## Abstract

In the last decades, various non-pharmacological solutions have been tested on top of medical therapy for the treatment of patients affected by refractory angina (RA). Among these therapeutics, neuromodulation, external counter-pulsation and coronary sinus constriction have been recently introduced in the guidelines for the management of RA in United States and Europe. Notably and paradoxically, although a consistent body of evidence has proposed cell-based therapies (CT) as safe and salutary for RA outcome, CT has not been conversely incorporated into current international guidelines yet. As a matter of fact, published randomized controlled trials (RCT) and meta-analyses (MTA) cumulatively indicated that CT can effectively increase perfusion, physical function and well-being, thus reducing angina symptoms and drug assumption in RA patients. In this review, we (i) provide an updated overview of novel non-pharmacological therapeutics included in current guidelines for the management of patients with RA, (ii) discuss the Level of Evidence stemmed from available clinical trials for each recommended treatment, and (iii) focus on evidence-based CT application for the management of RA.

## Introduction

Refractory angina (RA) is a clinical condition defined by the presence of persistent (≥3 months) symptoms of angina, according to the Canadian Cardiovascular Society (CCS) class, which is caused by untreatable coronary artery disease with objective evidence of reversible myocardial ischemia (1, 2). Clinical data Registry of the prevalence and incidence of RA remain limited and geographically clustered, making difficult a comprehensive evaluation of this clinical problem worldwide. One of the most widely recognized critical issues for the lack of epidemiologic data is the heterogeneous phenotype of patients labeled with a diagnosis of RA, which encompasses those with incomplete revascularization, unsuitable coronary anatomy, comorbidities, and other coronary disorders (3). The global prevalence of RA is increasing due to the growing prevalence of advanced aged population with coronary artery disease. According to the Heart Disease and Stroke Statistics-2019 (4), 9,400,000 patients are estimated to live with chronic angina and the proportion of these patients meeting the RA criteria lies in the esteem of about 7.5% (1, 5). On the basis of 1 million cardiac angiograms performed *per* year in the USA (6), the incidence of RA is then estimated to be 67,000 new cases/year (7).

The successful management of RA is often extremely challenging. At present, therapeutic options span from lifestyle modifications, state-of-the-art pharmacological therapy up to the most advanced mechanical revascularization solutions, with the main goal of improving prognosis, minimizing or abolishing symptoms and preventing episodes of angina (8–10). Nevertheless, it is important to underline that patients with persistent or recurrent chest pain despite optimal medical therapy frequently attend the general practitioner and/or the outpatient referrals and revisit hospital emergency departments, often undergoing repeated angiographic investigations. In this context, the social and economic burden for National Health Systems remains considerable due to the high rates of hospitalizations and multiple medications despite a limited quality of life. A recent Ontario-based study conservatively estimated the annual costs of angina-related disability (from a societal perspective including direct, indirect, and system costs) at $19,209 *per* patient (11).

Given these premises, novel treatments for RA in patients nonresponsive to standard pharmacologic therapies and not amenable to mechanical revascularization procedures are evermore needed. Notably, the one and only anti-anginal drug approved by the US Food and Drug Administration (FDA) in the last 20 years ranolazine (a selective inhibitor of the late sodium current—INaL—in cardiomyocytes) (12), was recently questioned in light of the uncertain evidence related to the safety and efficacy in reducing cardiovascular mortality, all-cause mortality, non-fatal acute myocardial infarction and frequency of angina (13).

It is worth to highlight that the long-term mortality of patients with RA is not as high as previously thought, reaching a 9-year rate of 28.4% (13). Therefore, the goal of novel therapies is primarily aimed at improving quality-of-life and chest pain relief rather than extending lifespan.

## Guidelines and Recommendations for Refractory Angina

Several innovative therapeutics have been developed to specifically address anginal symptoms. As suggested by Gallone et al. (3), these therapeutics can be classified as treatments targeting myocardial perfusion by (i) invasive/non-invasive interventions or treatments addressing neural processing and by (ii) chemical, mechanical or electrical means to interfere with pain signal. The former includes enhanced external counterpulsation (EECP) (14), coronary sinus reducer (CSR) (15), transmyocardial laser revascularization (TMLR) (16), extracorporeal shockwave myocardial revascularization (17), and cell-based applications (18). The latter comprises spinal cord stimulation (SCS) (19), cervico-thoracic stellate ganglion blockade/sympathectomy (20), and subcutaneous or transcutaneous electrical nerve stimulation (21).

Noteworthy, some of the above-mentioned technologies have already provided evidence of feasibility and clinically efficacy for RA patients qualified as “no option.” Notably, as the Level of Evidence supporting such advanced therapeutic strategies differs significantly and is constantly evolving as new evidence becomes available, guidelines are needed to incorporate such information. The most updated clinical practice guidelines for RA have been issued by the task forces of the American College of Cardiology/American Heart Association (ACC/AHA) in 2012 (22) and 2014 (23) and the European Society of Cardiology (ESC) in 2019 (2), respectively. In essence, these guidelines provide recommendations related to the treatment options available for RA based on a systematic review of the up-to-date evidence at the time of their publication. As usual, these recommendations are rely on Level of Evidence (from A to C) and class of recommendation (I, IIa, IIb, and III). Among the emerging non-pharmacological technologies, those listed in current United States (US) and Europe (EU) guidelines (Table 1) are the following: (1) EECP, (2) SCS, (3) CSR, and (4) TMLR. Cumulatively, EECP, are considered as treatments recommended for RA, even if with a relatively weak effectiveness level (class of recommendation IIb/Level of Evidence B). Conversely, TMLR is currently not recommended in EU (class of recommendation III/Level of Evidence A) (2).

**Table 1:** Level of evidence of non-pharmacological treatment options in the 2019 ESC and 2012–2014 ACC/AHA guidelines for refractory angina.

**Treatment strategy**	**2019 ESC guidelines**		**2012–2014 ACC/AHA guidelines**	
	**Class**	**Level of evidence**	**Class**	**Level of evidence**
Enhanced external counterpulsation	IIb	B	IIb	B
Spinal cord stimulation	IIb	B	IIb	C
Coronary sinus reducer	IIb	B	–	–
Transmyocardial laser revascularization	III	A	IIb	B

### Enhanced External Counterpulsation

The EECP is a non-invasive FDA approved therapy for patients with RA. The first model of external counterpulsation dates back to the 60's. The modern EECP, developed in 1983 (24), consists of three pairs of external cuffs compressing the calves, lower and upper thighs, which are inflated/deflated from distal to proximal according to the cardiac cycle. While in diastole the device aims to increase the retrograde aortic flow, improve coronary perfusion and venous return, in systole it reduces systemic vascular resistance, improve cardiac workload and systemic perfusion. The standard treatment protocol includes a total of 35 1-h sessions (5 days/week for 7 weeks). Two different and complementary mechanisms of action have been associated with the beneficial anti-ischemic effects of EECP therapy. Firstly, it was supported the concept that EECP, akin to a circulatory support, exerts central hemodynamic effects by improving coronary collateral growth and fractional flow reserve (i.e., oxygen supply) (25, 26). Secondly and more recently, researchers have focused on the direct and durable effect of EECP on the peripheral vasculature (i.e., oxygen demand). In particular, EECP has been shown to reduce arterial wall stiffness, promote peripheral artery flow-mediated dilation and improve shear stress, thus modulating the release of endothelial-derived vasoactive agents, pro-inflammatory cytokines, endothelial adhesion molecules and markers of lipid peroxidation (27–29).

The largest randomized controlled trial (RCT) aimed at evaluating the efficacy of EECP therapy in patients with RA (MUST-EECP trial) indicated that the application of EECP, when compared with a sham protocol (*n* = 59 treated patients vs. *n* = 65 controls), is safe with minor adverse events and provides clinical improvements in relation to the frequency of angina episodes, use of nitrates and time to exercise-induced ischemia. A number of smaller observational and randomized clinical trials (27–36) have generated three relevant meta-analyses (MTA) reporting positive results with regard to objective and subjective outcomes of angina (37–39). In particular, Qin et al. (37) showed a significant increase in myocardial perfusion, particularly in those patients who completed the entire 35 EECP sessions (pooled weighted mean difference from pre- to post-EECP: −0.19, 95% CI: −0.38 to 0.00, *p* = 0.049). However, as also declared by the authors, this study presented some limitations including the small sample size (*n* = 109 patients) and the high variability among imaging techniques applied (37). Other MTA found a reduction of at least 1 CCS functional class in 85% of patients treated with EECP (38, 39). Notably, some investigators confirmed the sustained benefit of EECP therapy for up to 5 years in relation to the frequency of angina episodes and major adverse cardiac events (MACE), although the results mainly stemmed from uncontrolled studies (34, 36, 40).

Based on these premises, the ACC/AHA and ESC guidelines concordantly recommend a class IIb/Level of Evidence B for EECP. However, it is important to highlight that, despite substantial evidence in its favor, EECP application has still not widely entered clinical practice since a number of critical issues and limitations remain unresolved, including the time-consuming protocol (1 h for 35 days), minor and major contraindications (e.g., coagulopathy, arrhythmias, peripheral artery, and venous disease), reimbursement issues and the lack of specialized centers.

### Spinal Cord Stimulation

Spinal cord stimulation (SCS) is a FDA-approved device conceived to alleviate chronic pain derived from various pathological conditions including chronic RA. The device consists in a programmable pulse-generator placed subcutaneously, below the left costal arch, and multipolar leads which are introduced under fluoroscopic guidance into the epidural space between the C7 and T4 level to obtain precordial pain relief. Although the standard protocol requires generally 1-h session, 3 times a day, the SCS device allows the modulation and the self-control of the stimulation based on the intensity of angina attacks. The precise mechanism by which SCS acts is still not fully understood. Its use was proposed for the first time on the basis of the “pain gate control” theory according to which impulses are transmitted in the nociceptive C-fibers of the central nervous system (41, 42). In patients with RA, SCS can provide dual beneficial effects: an analgesic effect by reduction of cardiac neuron activity following an ischemic attack, and a more debated anti-ischemic effect by adenosine-mediated coronary vasodilation and reduction of sympathetic tone (43–48). For example, the implantation of SCS device in RA patients has been associated with the improvement of myocardial ischemia tolerance, myocardial blood flow, and endothelium-mediated vasomotor function (48).

In the clinical setting of RA, spinal cord stimulation has been widely investigated in uncontrolled studies (48–54) or in comparison with various control treatments such as mechanical revascularization, standard-of-care or inactivated device (46, 55–57). Most of them reported positive results as regard to angina symptoms, quality of life, and acute hospital admissions. In particular, the ESBY trial, in which 53 RA patients receiving SCS were compared with 51 controls receiving coronary artery bypass grafting (CABG) for symptomatic indication “only”, demonstrated an equivalent effect of both treatments in terms of angina relief at 6 months (*p* < 0.0001); although the CABG group experienced higher exercise capacity and decreased ST-segment depression at follow-up (56). Moreover, the analysis of 121 patients enrolled in the European Angina Registry Link Study indicated a long-term efficacy of SCS implantation (mean 12.1 months) (49). Unfortunately, the STARTSTIM trial, which was designed to enroll a sufficient number of patients to support regulatory approval in the United States (by measuring the time to angina onset on standard exercise treadmill test at 6 months as primary endpoint), was prematurely stopped due to low recruitment rate (58). By merging the results of multiple clinical studies, five MTA and systematic reviews have been published so far (59–63). The comprehensive analysis of 14 studies which includes a total of 518 participants demonstrated that patients receiving SCS have longer exercise resistance (1.90 min, 95% CI: 1.71, 2.06), lower angina frequency (1.55 less daily; 95% CI: −1.75, −1.33), reduced nitrate consumption (1.54 less daily; 95% CI: −1.81, −1.26) and improved quality of life (95% CI: 10.76, 32.81; *p* < 0.0001) (59). These encouraging results were mitigated by other MTA which reported mild or small angina improvements (60, 63), also arising the problem of study interpretation due to the great variability in clinical trial designs (62). Although the safety profile appears to be satisfactory, a number of complications strictly related to the device implantation were reported and includes implant failure (49), lead displacement and superficial infections at the side of electrode insertion or pulse-generator (54). In essence, SCS in this clinical context does not seem to be an attractive area of investigation anymore if we look at the number of ongoing registered studies on clinicaltrials.gov. Consistently, the most recent ACC/AHA and ESC guidelines for the management of chronic stable angina made no change to recommendation for the use of SCS which remains Class IIb/Level of Evidence C in US (22) and Class IIb/Level of Evidence B in EU (2).

### Coronary Sinus Reducer

The coronary sinus reducer (CSR) is a relatively novel CE marked device designed to reduce disabling symptoms and improve quality-of-life of patients dealing with RA (15). It follows a long-standing concept of surgical narrowing of the coronary sinus proposed by Beck and colleagues between 1950's and 1960's (64). Basically, it is a balloon expandable stainless-steel mesh with the shape of an hourglass that is implanted percutaneously *via* the right jugular vein and works by creating a focal narrowing of the coronary sinus lumen. The subsequent elevated backward pressure in the coronary venous system leads to redistribution of blood flow from the less ischaemic subepicardium to the more ischaemic subendocardium. As therapy for “no option” RA patients, CSR was proposed for the first time in 2007 (65). Although based on registries and open-label/uncontrolled trials (66–70), the majority of published studies provided evidence of angina relief showing a 70–80% rate of treatment-responders (15). In this context, the largest available study is the COSIRA trial (COronary SInus Reducer for treatment of refractory Angina) in which 52 RA patients were allocated to CSR implantation and 52 to a sham procedure (71). After 6 months from the device implantation the 71% of treated patients experienced an improvement of at least 1 CCS class as compared with 42% of controls (*p* = 0.003). In addition, a *post-hoc* efficacy analysis revealed a significant between-group differences in exercise time improvement (+27.9, 95% CrI = 2.8–59.8%) and quality of life (stability +11.2 points, 95% CrI = 3.3–19.1; perception +11.0, 95% CrI = 3.3–18.7) (72). Consistently, a systemic review, by combining the results of six studies and 196 patients, showed that CSR significantly improves CCS angina class (from 3.2 at baseline to 1.9 after a mean follow-up of 8.6 months) (73). On the other hand, this work provides some interesting insights about the CSR safety profile. Indeed, a 2% implantation failure rate (e.g., unsuitable coronary sinus or valvular anatomy) as well as a 2.6% of short-term complications (e.g., migration, hematoma, non-ST elevation myocardial infarction) were documented (73). It is worth to highlight that 20–30% of patients are still deemed non-responders for reasons still not fully elucidated. In the attempt to predict responsiveness to CSR implantation, Baldetti et al. (74) measured the differential pressure between baseline right atrial pressure and coronary sinus systolic pressure in the context of coronary sinus balloon occlusion showing that the patient group having a developed accessory venous drainage systems had lower anti-ischemic effects due to preserved alternative coronary venous outflow.

Interestingly, a health technology analysis on CSR device for RA patients was recently made available (75). Results confirmed the positive impact of CSR regarding both objective and subjective endpoints of ischemia (i.e., Seattle Angina Questionnaire score, dobutamine echocardiography, thalium single-photon emission computed tomography perfusion studies, and 6-min-walk test and myocardial perfusion reserve index). Yet, these findings should be considered with caution since the lack of internal validity of included studies may have undermined the positive results. More definitive indications will likely come from the on-going clinical investigations evaluating (i) the long-term safety and benefit of CSR therapy (NCT02710435), (ii) the objective improvement of CSR implantation in terms of exertional capacity and myocardial reversible ischemia (NCT04121845). According to the abovementioned evidence, CSR device received class IIb recommendation and Level of Evidence B from the 2019 ESC guidelines. In US, CSR was granted with a “Breackthrough Designation” by the FDA in 2018 based on the “orphan” need of this population but additional data are required to enter into US guidelines.

### Transmyocardial Laser Revascularization

The transmyocardial laser revascularization (TMLR) technique uses FDA approved laser ablation (i.e., carbon dioxide, holmium: yttrium-aluminum-garnet [Ho:YAG] or XeCL excimer) to create transmural channels in targeted ischemic regions of myocardium to restore myocardial perfusion. The beneficial effect of TMLR has been ascribed to two principal mechanisms; sympathetic denervation that acts for the acute clinical benefits and angiogenesis responsible for the long-term benefits. The procedure was performed either surgically or percutaneously.

The surgical approach *via* thoracotomy or sternotomy allows direct position of a laser device on the epicardial surface of the left beating ventricle and the delivery of ~1 mm transmural laser channels from the epicardium to the endocardium. In the past years surgical TMLR for RA was investigated either as a stand-alone therapy for patients not suitable to further revascularization procedures (76–81) or in combination with CABG for those patients who would be incompletely revascularized with CABG alone (82–86). In particular, Allen et al. (80) demonstrated the superiority of sole TMLR vs. best medical treatment in improving classes of angina (*p* < 0.001), survival free from cardiac events (*p* < 0.001), exercise tolerance (*p* = 0.05), and quality-of-life scores (*p* = 0.003). However, a similar study design did not demonstrate objective difference in exercise time and walking distance, although improvements in angina were showed (81). Regarding TMLR combined with CABG, a multicenter, randomized, prospective study enrolling 266 RA patients blinded to treatment arm indicated that CABG *plus* TMLR is more effective in lowering operative mortality, post-operative inotropic support and short-term MACE compared to CABG alone (86). Furthermore, these results were confirmed after a 5-year follow-up, showing a sustained reduction of recurrent severe angina in the CABG *plus* TMLR group, although the survival rate was not different (82).

The percutaneous approach has been proposed as a less invasive strategy taking the advantage of commercialized catheters designed for positioning an optical fiber coupled to a laser. This application was tested in multiple unblinded studies with discordant results (77, 87–90). Of note, the “DMR In Regeneration of Endomyocardial Channels—DIRECT” Trial, which was the first and only RCT study with blinded patients and outcome assessors, reported essentially negative results in terms of exercise duration, angina symptoms, and myocardial perfusion scores (91).

In a limited number of pilot experiences, TMLR was used, either surgically or percutaneously, as an adjunctive therapy to cell therapy with the rationale to boost the angiogenic response (92–96).

By combining all these important studies, the Cochrane reviewers provided evidence of higher early post-operative mortality in patients treated with TMLR compared to standard medical therapy (pooled OR was 3.76, 95% CI: 1.63–8.66) (97).

On these bases, surgical TMLR and percutaneous TMLR are not recommended in EU (Class III recommendation) while in US a Class IIb/Level of Evidence B recommendation was given in the last 2012 ACC/AHA guideline.

## The Case of Cell Therapy

**Figure 1:**
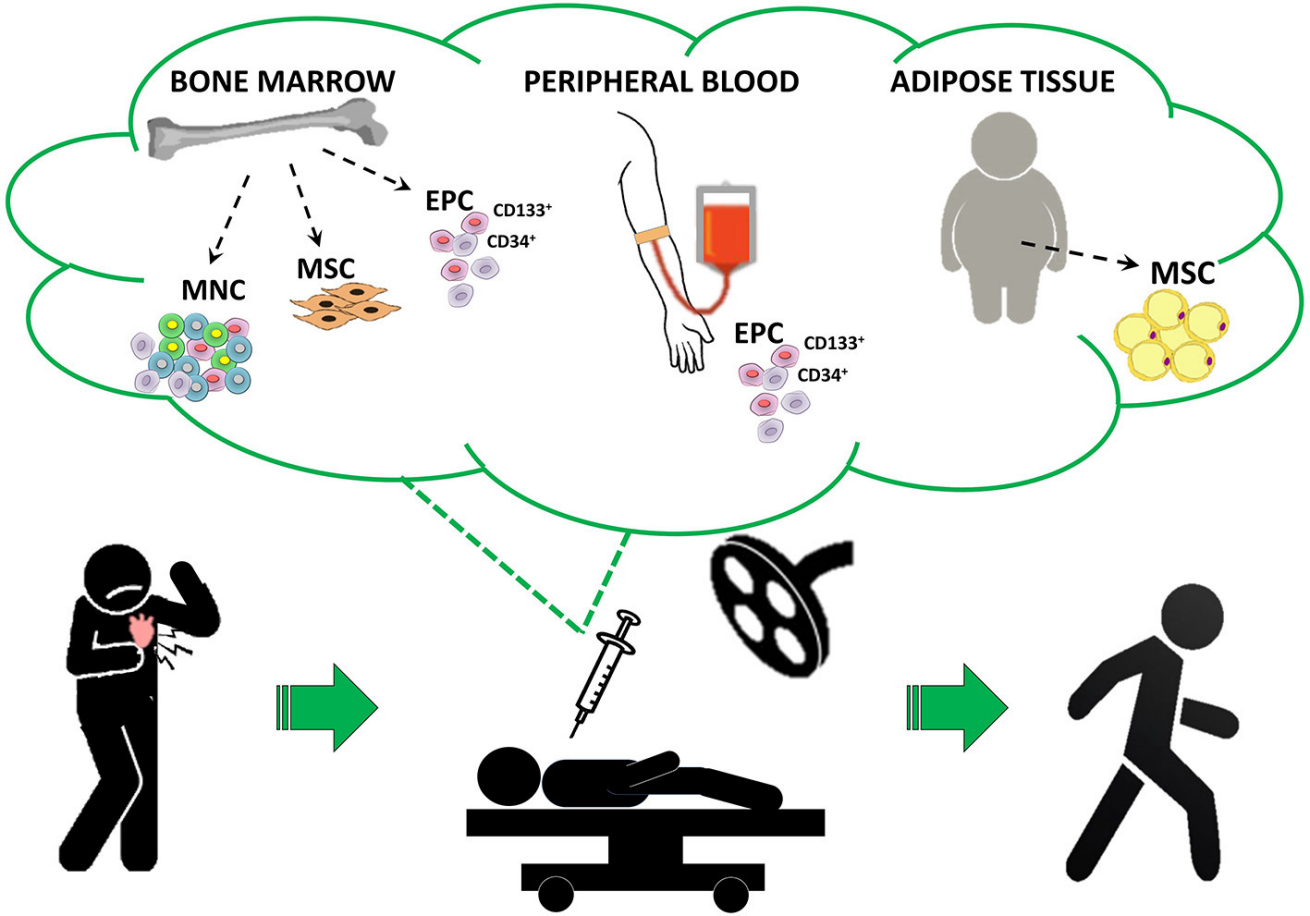
Proposed cell therapy approach for refractory angina patients. The figure represents the ideal in-hospital protocol of different cell-based therapies for RA following a gold standard approach. EPC, endothelial progenitor cells; MNC, mononuclear cells; MSC, mesenchymal stem cells.

Cell-based therapies (CT) for heart diseases have been extensively investigated over the last 20 years and, despite a number of methodological limitations regarding both cell therapeutics and patient profile might have influenced clinical outcomes (98), RA appears the cardiac conditions in which CT has shown the most promising results. Indeed, a consistent body of evidence (RCT and MTA) cumulatively indicated that CT is safe and can effectively increase physical function and well-being by reducing angina symptoms and drug assumption in the absence of relevant side effects (18). Different pro-angiogenic cells were administered in an autologous setting, including unfractioned bone marrow (BM)-derived mononuclear cells (MNC) (99, 100), selected endothelial progenitors (i.e., CD34^+^ and CD133^+^ cells) derived from BM or peripheral blood (101–104) or mesenchymal stem cells derived from BM (105) or adipose tissue (106–108) (Figure 1). In addition to pilot and proof-of-concept clinical studies, a significant proportion of published trials may be categorized as phase II RCT (100, 101, 109–111). In particular, the ACT34-CMI trial, by enrolling 167 RA patients to receive intramyocardial injection of BM-derived CD34^+^ cells (0.1 × 10^6^ or 0.5 × 10^6^ cells/Kg) or placebo, demonstrated the superiority of CD34^+^ cells vs. placebo in improving exercise tolerance (*p* = 0.01) and weekly angina frequency (*p* = 0.02), especially for the group that received 0.1 × 10^6^ CD34^+^ cells/Kg (111). The 2-year follow-up confirmed the persistence of clinical effects along with a trend of reduction in MACE (112). Similarly, positive results were observed in the study of van Ramshorst and coworkers in which the treatment with 100 × 10^6^ autologous BM-derived MNC is associated with a significant improvement of myocardial perfusion at single-photon emission computed tomography (*p* = 0.001) and CCS class (*p* = 0.001), in parallel with a modest LVEF amelioration at MRI (~3%) after 6 months of follow-up (100).

These favorable results encouraged the initiation of three large phase III RCT. However, none of them can be considered conclusive due to early termination for (i) sponsor strategic reasons (RENEW study (102)), slow recruitment rate (REGENT-VSEL trial (103)), and procedure-related issues (ATHENA trial (113)). More in details, the phase III RENEW trial was designed to definitely assess the efficacy of intramyocardial injection of autologous CD34^+^ cells in 444 “no option” RA patients. Unfortunately, results were available for only 112 patients suggesting, in accordance with earlier phase studies, a greater exercise capacity and a dramatic reduction in angina frequency in CT treated patients (102). Conversely, the recent sub-analysis of the REGENT-VSEL trial did not demonstrate a statistical difference of quality of life and clinical symptoms in patients receiving CD133^+^ cells compared with those receiving placebo (114).

To combine multiple clinical research results, six MTA were conducted so far and the cumulative results on CT safety and efficacy have been shown (115–120), among which the most updated are herein presented. The work of Shah et al. (115), based on 10 RCT including 658 patients with 6- to 24-month follow-up, represent the most comprehensive MTA on this topic. In particular, CT in RA patients determined an improvement in CCS class (risk ratio (RR) [95%CI]: 1.53 [1.09, 2.15], *p* = 0.013), exercise capacity (standard mean difference (SMD) [95%CI]: 0.56 [0.23, 0.88], *p* = 0.001), and a reduction in angina frequency (SMD [95%CI]: −1.21 [−2.40, −0.02], *p* = 0.045). Moreover, authors highlighted that CT has positive effects on myocardium by reducing perfusion defects (SMD [95%CI]: −0.70 [−1.11, −0.29], *p* = 0.001) and improving LVEF (SMD [95%CI]: 0.64 [0.27, 1.00], *p* = 0.001). The risk of all-cause mortality was similar in patients treated with CT or placebo (*p* = 0.121). It is important to point out that such results, although promising, derived from the pooled effect of different cell products and, thus, cannot be deemed conclusive but only hypothesis-generating.

In this regard, a less comprehensive, but more focused, MTA published by Velagapudi et al. (117) provided strong evidence supporting beneficial effect of intramyocardial delivery of CD34^+^ cell-based therapy in RA and a rationale for a definitive Phase III RCT. As for safety, the risk of MI and stroke did not differ in patients treated with CD34^+^ cells with respect to placebo (odd ratio (OR) [95%CI]: 0.77 [0.36, 1.63] and 0.50 [0.08, 3.06], respectively), but, in return, the overall risk of mortality was significantly lower in CD34^+^ cell than in placebo group (0.24 [0.08, 0.73], *p* = 0.01) (117). Finally, the most updated systematic review further confirmed that CT in RA patients entails lower incidence of MACE (OR [95%CI]: 0.41 [0.25, 0.70], *p* < 0.0001) and all-cause mortality (0.24 [0.10, 0.60], *p* = 0.002) respect to placebo/controls (116). Interestingly, the subgroup analysis revealed that the favorable outcome in the pooled analysis is primarily driven by data derived from clinical studies with CD34^+^ cells which embody the largest patient cohort (74%) (116). Recently, the retrospective analysis of phase I/IIa, phase II ACT-34 and phase III RENEW was published showing that RA patients who received CD34^+^ cell therapy experienced the reduction of hospitalizations, cardiac procedures, and health care expenditures in the first year following treatment compared to the year prior (121).

## Discussion and Conclusions

**Figure 2:**
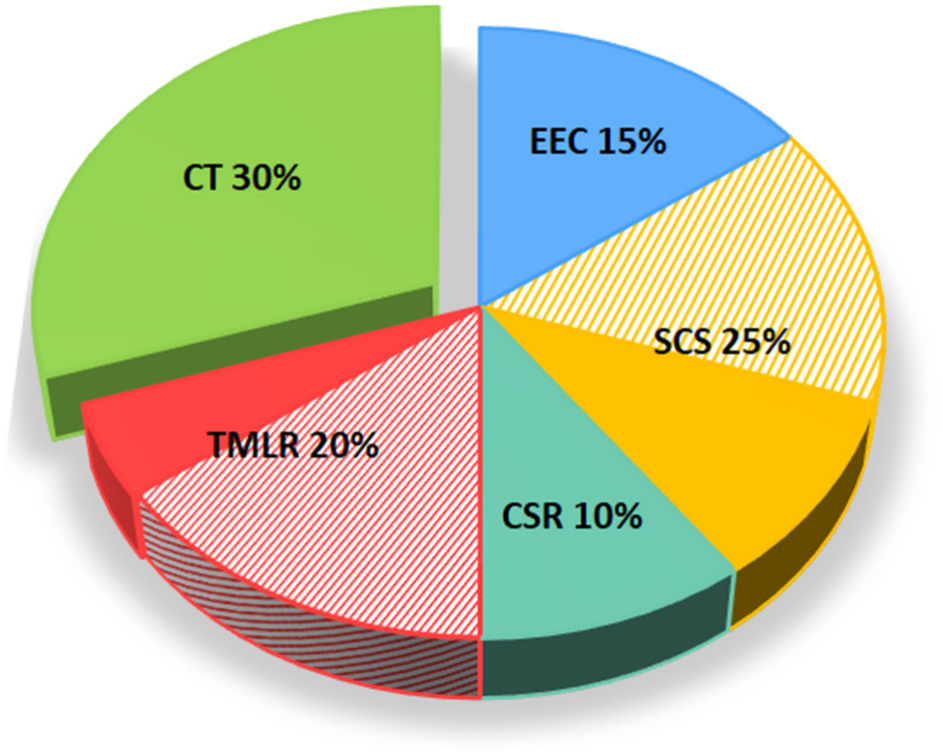
Meta-analyses and outcomes of non-pharmacological treatment options for refractory angina as per guidelines. The figure depicts the number and outcomes of available MTA for each non-pharmacological treatment options for RA including EEC, SCS, and CSR, in addition to CT. Positive MTA are represented with full color while those negatives are depicted with stripes. CSR, coronary sinus reducer; CT, cell-based therapy; EECP, enhanced external counterpulsation; MTA, meta-analysis; RA, refractory angina; SCS, spinal cord stimulation.

The management of RA patients is still challenging, as demonstrated by the most recent reviews on the topic (122–124). After exhausting traditional medical therapies, the options for RA are very limited with EECP, SCS and CSR being the only recommended approaches (2, 23). In essence, to date we do not have a definitive answer on the best non-pharmacological treatment strategy for RA because, as shown in Table 2, each comes with its own advantages and disadvantages. Nevertheless, it is clearly evident that CT for this specific cardiac condition has all the features to be ultimately considered in the international guidelines. Indeed, a substantial body of clinical evidence, by means of RCT and MTA, indicates CT as a viable therapeutic option for RA, which appears a favorable target for the first introduction of CT in the clinical arena. As depicted in Figure 2, a number of MTA have been conducted in the past years to address the efficacy and safety of emerging non-pharmacological treatment options for RA, of which three for EECP, five for SCS, two for CSR, four for TMLR, and six for CT (see Supplementary Table 1 for evidence supporting Figure 2). As for SCS and TMLR, evidence arising from MTA is mixed. On the contrary, MTA for CT are the most represented and yielded 100% positive outcomes. Despite this, CT for RA has not yet been incorporated into current guidelines and relegated as a “potential treatment option.”

**Table 2:** Non-pharmacological treatment options in the 2019 ESC and 2012–2014 ACC/AHA guidelines for refractory angina vs. cell therapy.

**Treatment strategy**		**Proposed principle of action**	**Effectiveness**	
			**Pros**	**Cons**
Recommended	Enhanced external counterpulsation	Improved venous return and coronary perfusion in diastole, decreased workload in systole	+++ Improved indices of angina, myocardial perfusion, exercise capacity, and MACE	Time-consuming protocol, minor, and major contraindications (e.g., coagulopathy, arrhythmias, peripheral artery, and venous disease)
	Spinal cord stimulation	Reduction of cardiac neuron activity and sympathetic tone, anti-ischemic effect by adenosine-mediated coronary vasodilation	+/– Improved indices of angina and exercise capacity	Invasive, surgical complications (e.g., implant failure, lead displacement, and infections)
	Coronary sinus reducer	Coronary blood flow redistribution	+++ Improved indices of angina, myocardial perfusion, and exercise capacity	Invasive, surgical complications (e.g., implant failure, migration, hematoma, NSTEMI)
	Transmyocardial laser revascularization	Sympathetic denervation and angiogenesis	No effect	Invasive, post-procedural higher mortality
Not yet recommended	Cell therapy	Angiogenesis and cardioprotection	+++ Improved indices of angina, myocardial perfusion, exercise capacity, and MACE	Invasive, surgical complications (e.g., hematoma, bleeding, and arrhythmias)

On top of guidelines, the introduction of CT into the therapeutic armamentarium of cardiologists needs to match the regulatory framework of advanced medicinal products. In addition, it is important to point out that CT (differently from other non-pharmacological technologies described above) cannot be conceived as a unique therapeutic agent, but as a wide spectrum of highly innovative products which have to ensue specific development plans and regulatory pathways.

In this perspective, promising developments are expected from the CD34^+^ cell technology which has recently received a “Regenerative Medicine Advanced Therapy Designation” by the FDA to expedite the approval for use in no-option RA (125). In summary, we believe that the scientific and clinical framework is mature enough for the introduction in the international guidelines of the first biological product to cure RA.

## Author Contributions

BB, ER, and EG analyzed the studies and wrote the manuscript. GP conceived and wrote the manuscript. All authors have read and approved the final manuscript and agreed to be personally accountable for the author's own contributions.

## Conflict of Interest

EG is Chief Technology & Operating Officer of Oloker Therapeutics S.r.l. GP is Chairman of the Advisory Board of Oloker Therapeutics S.r.l. The remaining authors declare that the research was conducted in the absence of any commercial or financial relationships that could be construed as a potential conflict of interest.

## Publisher's Note

All claims expressed in this article are solely those of the authors and do not necessarily represent those of their affiliated organizations, or those of the publisher, the editors and the reviewers. Any product that may be evaluated in this article, or claim that may be made by its manufacturer, is not guaranteed or endorsed by the publisher.

## Abbreviations

ACC: American College of Cardiology
AHA: American Heart Association
BM: Bone marrow
CABG: Coronary artery bypass grafting
CCS: Canadian Cardiovascular Society
CSR: Coronary Sinus Reducer
EECP: Enhanced external counterpulsation
ESC: European Society of Cardiology
FDA: Food and Drug Administration
MACE: Major adverse cardiac events
MNC: Mononuclear cells
MTA: Meta-analysis
RA: Refractory Angina
RCT: Randomized controlled trial
SCS: Spinal Cord Stimulation
TMLR: Transmyocardial laser revascularization.
