# Supplementary material for: Son of a Lesser God: The Case of Cell Therapy for Refractory Angina
Source: Front Cardiovasc Med. 2021 Sep 6;8:709795. doi: 10.3389/fcvm.2021.709795 (PMC8450394; doi:10.3389/fcvm.2021.709795)
Supplement: Supplementary file 1 [file Table_1.DOCX]

Supplementary Material

# Supplementary Table 1

**Meta-analyses and outcomes of** **non-pharmacological treatment options for refractory angina as per EU and US-guidelines.**

| **MTA Reference** | **Positive** | **Negative/Neuter** |
| --- | --- | --- |
| ***Enhanced external counterpulsation*** | | |
| Quin *et al.* [1] | X | - |
| Shah *et al.* [2] | X | - |
| Zhang *et al.* [3] | X | - |
| ***Spinal cord stimulation*** | | |
| Imran *et al.* [4] | X | - |
| Pan *et al.* [5] | - | X |
| Taylor *et al.* [6] | X | - |
| Tsigaridas *et al.* [7] | X | - |
| Wang *et al.* [8] | - | X |
| ***Coronary sinus reducer*** | | |
| Bazoukis *et al.* [9] | X | - |
| Stanak et al. [10] | X | - |
| ***Transmyocardial laser revascularization*** | | |
| Cheg *et al.* 2006 [11] | X | - |
| McGillion *et al.* 2010 [12] | - | X |
| Briones *et al.* 2009 [13] | - | X |
| Liao et al. 2005 [14] | - | X |
| ***Cell therapy*** | | |
| Shah *et al.* [15] | X | - |
| Jones *et al.* [16] | X | - |
| Velagapudi *et al.* [17] | X | - |
| Khan *et al.* [18] | X | - |
| Li *et al.* [19] | X | - |
| Fisher *et al.* [20] | X | - |

**Supplementary References**

1. Qin X, Deng Y, Wu D, Yu L, Huang R: **Does Enhanced External Counterpulsation (EECP) Significantly Affect Myocardial Perfusion?: A Systematic Review & Meta-Analysis**. *PLoS One* 2016, **11**(4):e0151822.

2. Shah SA, Shapiro RJ, Mehta R, Snyder JA: **Impact of enhanced external counterpulsation on Canadian Cardiovascular Society angina class in patients with chronic stable angina: a meta-analysis**. *Pharmacotherapy* 2010, **30**(7):639-645.

3. Zhang C, Liu X, Wang X, Wang Q, Zhang Y, Ge Z: **Efficacy of Enhanced External Counterpulsation in Patients With Chronic Refractory Angina on Canadian Cardiovascular Society (CCS) Angina Class: An Updated Meta-Analysis**. *Medicine (Baltimore)* 2015, **94**(47):e2002.

4. Imran TF, Malapero R, Qavi AH, Hasan Z, de la Torre B, Patel YR, Yong RJ, Djousse L, Gaziano JM, Gerhard-Herman MD: **Efficacy of spinal cord stimulation as an adjunct therapy for chronic refractory angina pectoris**. *Int J Cardiol* 2017, **227**:535-542.

5. Pan X, Bao H, Si Y, Xu C, Chen H, Gao X, Xie X, Xu Y, Sun F, Zeng L: **Spinal Cord Stimulation for Refractory Angina Pectoris: A Systematic Review and Meta-analysis**. *Clin J Pain* 2017, **33**(6):543-551.

6. Taylor RS, De Vries J, Buchser E, Dejongste MJ: **Spinal cord stimulation in the treatment of refractory angina: systematic review and meta-analysis of randomised controlled trials**. *BMC Cardiovasc Disord* 2009, **9**:13.

7. Tsigaridas N, Naka K, Tsapogas P, Pelechas E, Damigos D: **Spinal cord stimulation in refractory angina. A systematic review of randomized controlled trials**. *Acta Cardiol* 2015, **70**(2):233-243.

8. Wang S, Li Q, Fang H, Yang H, Su D, Tao YX, Wang Z, Wang X, Yang Z: **Spinal cord stimulation versus other therapies in patients with Refractory Angina: A meta-analysis**. *Transl Perioper Pain Med* 2017, **2**(1):31-41.

9. Bazoukis G, Brilakis ES, Tse G, Letsas KP, Kitsoulis P, Liu T, Baranchuk A, Sideris A, Tsioufis C, Stavrakis S: **The efficacy of coronary sinus reducer in patients with refractory angina-A systematic review of the literature**. *J Interv Cardiol* 2018, **31**(6):775-779.

10. Stanak M, Rothschedl E, Szymanski P: **Coronary Sinus Reducing Stent for the Treatment of Refractory Angina Pectoris: A Health Technology Assessment**. *Med Devices (Auckl)* 2020, **13**:259-276.

11. Cheng D, Diegeler A, Allen K, Weisel R, Lutter G, Sartori M, Asai T, Aaberge L, Horvath K, Martin J: **Transmyocardial laser revascularization: a meta-analysis and systematic review of controlled trials**. *Innovations (Phila)* 2006, **1**(6):295-313.

12. McGillion M, Cook A, Victor JC, Carroll S, Weston J, Teoh K, Arthur HM: **Effectiveness of percutaneous laser revascularization therapy for refractory angina**. *Vasc Health Risk Manag* 2010, **6**:735-747.

13. Briones E, Lacalle JR, Marin-Leon I, Rueda JR: **Transmyocardial laser revascularization versus medical therapy for refractory angina**. *Cochrane Database Syst Rev* 2015(2):CD003712.

14. Liao L, Sarria-Santamera A, Matchar DB, Huntington A, Lin S, Whellan DJ, Kong DF: **Meta-analysis of survival and relief of angina pectoris after transmyocardial revascularization**. *Am J Cardiol* 2005, **95**(10):1243-1245.

15. Shah R, Latham SB, Khan SA, Shahreyar M, Hwang I, Jovin IS: **A comprehensive meta-analysis of stem cell therapy for chronic angina**. *Clin Cardiol* 2018, **41**(4):525-531.

16. Jones DA, Weeraman D, Colicchia M, Hussain MA, Veerapen D, Andiapen M, Rathod KS, Baumbach A, Mathur A: **The Impact of Cell Therapy on Cardiovascular Outcomes in Patients With Refractory Angina**. *Circ Res* 2019, **124**(12):1786-1795.

17. Velagapudi P, Turagam M, Kolte D, Khera S, Hyder O, Gordon P, Aronow HD, Leopold J, Abbott JD: **Intramyocardial autologous CD34+ cell therapy for refractory angina: A meta-analysis of randomized controlled trials**. *Cardiovasc Revasc Med* 2019, **20**(3):215-219.

18. Khan AR, Farid TA, Pathan A, Tripathi A, Ghafghazi S, Wysoczynski M, Bolli R: **Impact of Cell Therapy on Myocardial Perfusion and Cardiovascular Outcomes in Patients With Angina Refractory to Medical Therapy: A Systematic Review and Meta-Analysis**. *Circ Res* 2016, **118**(6):984-993.

19. Li N, Yang YJ, Zhang Q, Jin C, Wang H, Qian HY: **Stem cell therapy is a promising tool for refractory angina: a meta-analysis of randomized controlled trials**. *Can J Cardiol* 2013, **29**(8):908-914.

20. Fisher SA, Doree C, Brunskill SJ, Mathur A, Martin-Rendon E: **Bone Marrow Stem Cell Treatment for Ischemic Heart Disease in Patients with No Option of Revascularization: A Systematic Review and Meta-Analysis**. *PLoS One* 2013, **8**(6):e64669.
